# Supplementary material for: Enhanced production of mesencephalic dopaminergic neurons from lineage-restricted human undifferentiated stem cells
Source: Nat Commun. 2023 Dec 5;14:7871. doi: 10.1038/s41467-023-43471-0 (PMC10698156; doi:10.1038/s41467-023-43471-0)
Supplement: Supplementary file 1 — Supplementary Information [file 41467_2023_43471_MOESM1_ESM.pdf]

## SUPPLEMENTARY INFORMATION

# Enhanced Production of Mesencephalic Dopaminergic Neurons from Lineage-Restricted Human Undifferentiated Stem Cells.

Muyesier Maimaitili[1,2,†], Muwan Chen[1,2,†], Fabia Febbraro[2,3], Ekin Ucuncu[1,2], Rachel Kelly[1,2], Jonathan C. Niclis[4], Josefine Rågård Christiansen[4], Noëmie Mermet-Joret[1,5,6], Dragos Niculescu[1,2,6], Johanne Lauritsen[1,2], Angelo Iannielli[7,8], Ida Hyllen Klæstrup[1,2], Uffe Birk Jensen[2,3], Per Qvist[2,9,10,11], Sadegh Nabavi[1,5,6], Vania Broccoli[7,8], Anders Nykjær[1,2,6], Marina Romero-Ramos[1,2], Mark Denham[1,2,*]

Summary of content:

**Supplementary Figures**

Supplementary Figure 1: Generation of *GBX2* knockout cell line.
Supplementary Figure 2: Generation of 4X knockout from H9 cell line.
Supplementary Figure 3: Additional data for Fig. 1, 32 day differentiation.
Supplementary Figure 4: Additional data for Fig. 2, quadruple FACS panel data.
Supplementary Figure 5: NGS-sequencing data for 4X-H1-NC1, 4X-GBA-C7, and 4X-GBA-C8.
Supplementary Figure 6: Additional data for Fig. 2, DIV16 and 30 differentiation images and quantification data.
Supplementary Figure 7: Additional data for Fig. 3, single-cell data at DIV16.
Supplementary Figure 8: Expression of HOX genes at DIV 16.
Supplementary Figure 9: Additional data for Fig.3-4, differentiation under hindbrain condition at DIV16, 62, and 83.
Supplementary Figure 10: Additional data for Fig. 4, single-cell data at DIV62.
Supplementary Figure 11: Additional data for Fig. 6, First *in vivo* round.
Supplementary Figure 12: Additional data for Fig. 7, Second *in vivo* round.

**Supplementary Tables**

Supplementary Table 1: Guide strand target sequence and assessment of off-target activity in $GBX^{-/-}$ and 4X cell line.
Supplementary Table 2: QPCR primers
Supplementary Table 3: Nanostring probes

# Supplementary Figure 1

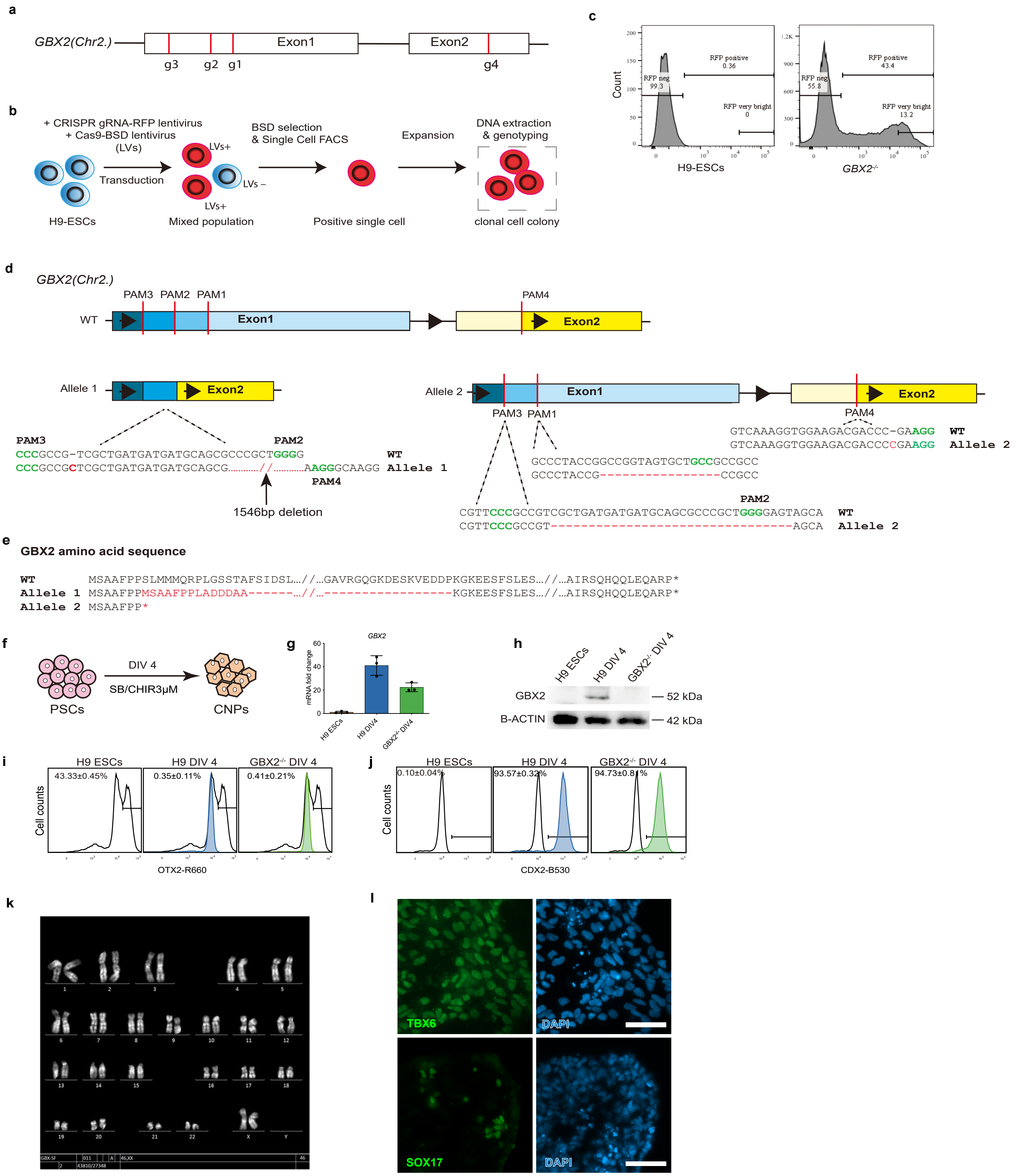


**Supplementary Figure 1: Generation of *GBX2*$^{-/-}$ cell line.**

**a**, Schematic diagram of the *GBX2* locus, showing the guide RNA-targeted sites (g1-g4) in red lines. **b**, Schematic overview of the CRISPR-Cas9 and single-cell-clonal selection strategy used to generate *GBX2*$^{-/-}$ cell line. **c**, Single-cell FACS sorting of the transduced cells based on the RFP signal intensity. H9 hESC line is used as a negative gating control for RFP. **d**, Mutational analysis by whole exome sequencing revealed biallelic indels mutation in the targeted *GBX2* loci. **e**, Predicted protein sequence of the mutant alleles. The out-of-frame sequences are shown in red. **f**, Schematic diagram of the differentiation protocol used to validate the loss of GBX2 in *GBX2*$^{-/-}$ cell line. **g**, RNA expression analysis of *GBX2* in H9 ESCs, H9 and *GBX2*$^{-/-}$ cells at DIV4, n = 3 independent experiments (mean and error bars = S.D). **h**, Western blot analysis of GBX2 in H9 ESCs and caudal neural progenitors (CNPs) at DIV4, showing no detectable GBX2 in *GBX2*$^{-/-}$ cell line. β-actin shown as loading control (n = 2 independent experiments). H9 ESCs are used as a negative control in **(g)** and **(h)**. **i-j**, Flow cytometer analysis of the percentage of OTX2-postive **(i)** , CDX2-positive **(j)** cells among H9 and *GBX2*$^{-/-}$ at DIV4. n = 3 biological replicates. Blasticidin (BSD). **k,** Karyotype analysis of GBX2-/- cell line, n = 5 karyotypes. **l**, Differentiation into mesoderm (TBX6) and endoderm (SOX17) lineages, n = 3 biological replicates  (scale bar: 50μm).

# Supplementary Figure 2

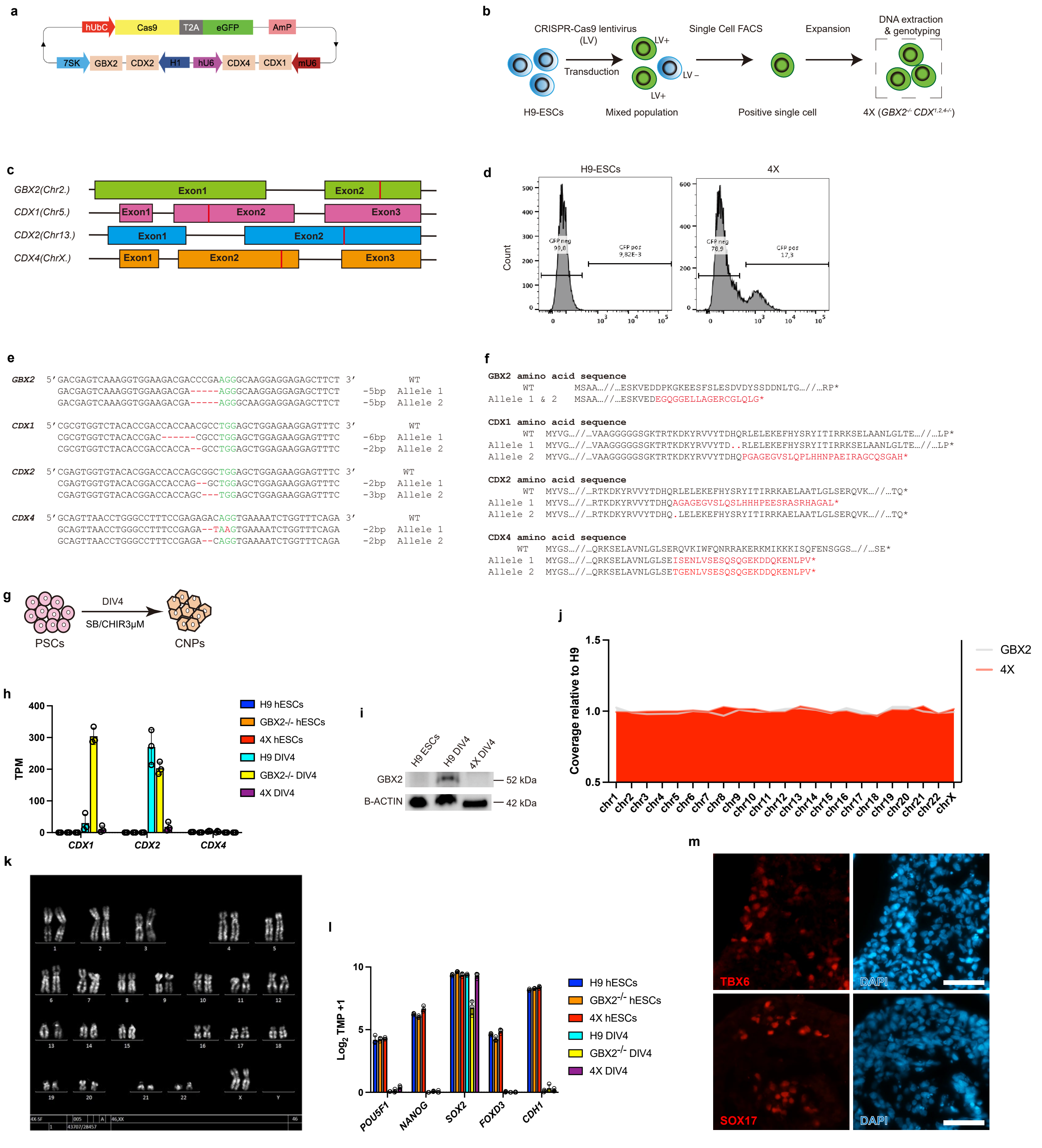


**Supplementary Figure 2: Generation of 4X cell line.**

**a**, Schema of the Cas9 lentiviral construct used to target *GBX2, CDX1, CDX2* and *CDX4.* **b**, Schematic overview of the single cell clonal selection of CRISPR-modified hESCs. **c**, Schematic diagram of the Cas9-targeted genes. The guide RNA-targeted sites (g1-g4) are indicated by red lines. **d**, Single-cell FACS sorting of the transduced cells based on the GFP signal intensity. H9 hESC line is used as a negative gating control for GFP. **e**, Mutational analysis by whole exome sequencing identified biallelic mutations in the targeted genes. **f**, Predicted protein sequence of the mutant alleles. The out-of-frame sequences are shown in red. **g**, Schematic diagram of the differentiation protocol used to validate the loss of CDX2 and GBX2 in 4X cell line. **h**, RNA expression analysis of CDX1, *CDX2, and CDX4* in H9, GBX2$^{-/-}$, and 4X cells at DIV0 and DIV4 (NMPs), n = 3 independent experiments, mean and error bars = S.D. **i**, Western blot analysis of GBX2 in H9 ESCs and caudal neural progenitors at DIV4, showing no detectable GBX2 protein in 4X cell line. β-actin shown as loading control (n = 2 independent experiments). H9 ESCs are used as a negative control. **j**, Copy number variation analysis of exome sequencing data for H9, GBX2$^{-/-}$, and 4X. **k,** Karyotype analysis of 4X cell line, n = 5 karyotypes. **l**, Expression of pluripotent markers in cell lines H9, GBX2$^{-/-}$, 4X at the undifferentiated state and after 4 days differentiation with SB, CHIR. n = 3 independent experiments, mean and error bars = S.D. **m**, Differentiation of 4X into mesoderm (TBX6) and endoderm (SOX17) lineages, n = 3 biological replicates (scale bar: 50μm).

# Supplementary Figure 3

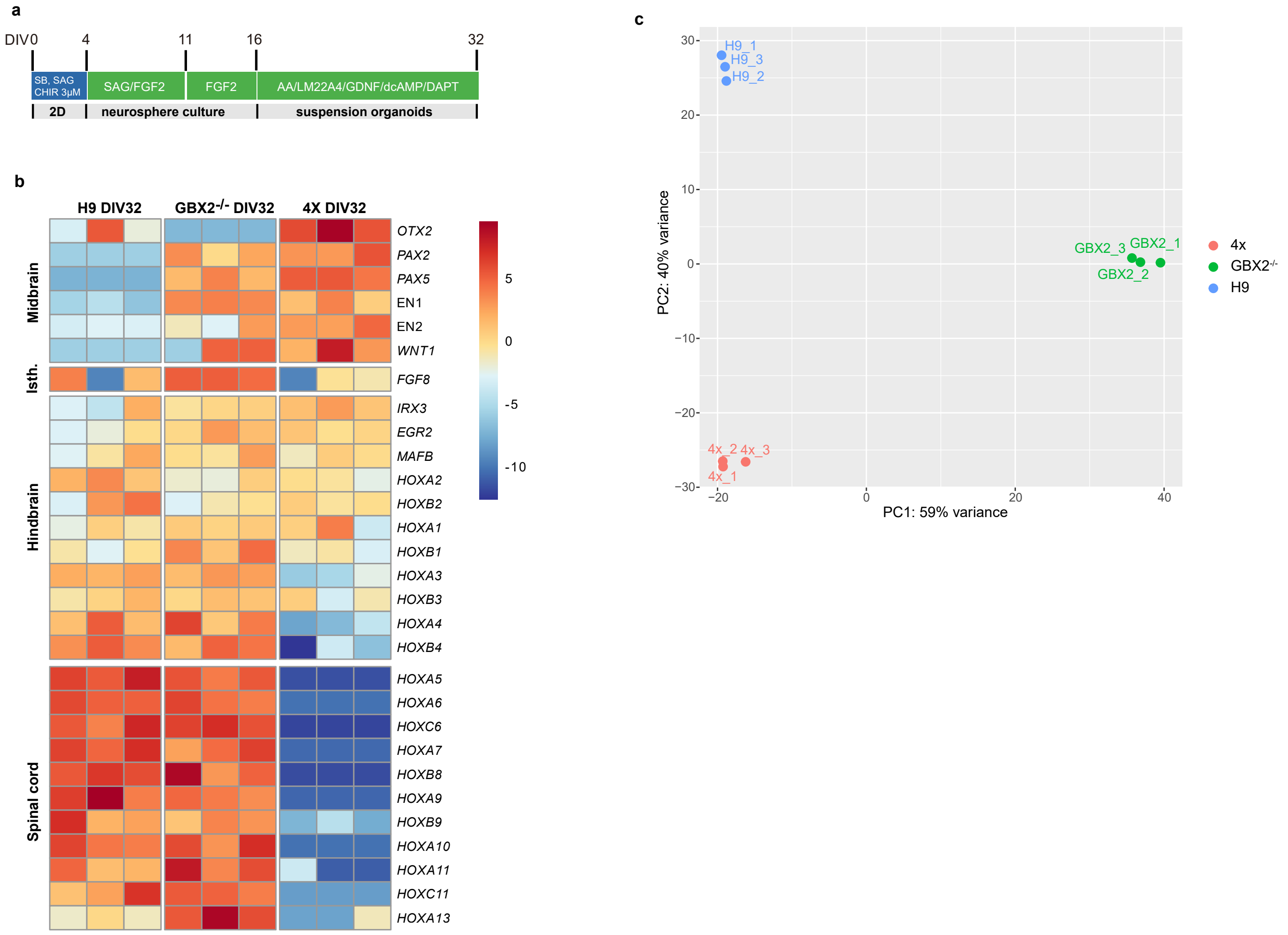


**Supplementary Figure 3: Differentiation of H9, GBX2$^{-/-}$, and 4X to 32 days**
**a**, Diagram of differentiation protocol. isth. = isthmic organizer. **b**, Heatmap of genes expressed along the anterior and posterior axis. **c**, PCA plot of RNA sequencing data from H9, GBX2$^{-/-}$, and 4X. The data presented are from n = 3 biological replicates, with each replicate containing 9 spheres.

Supplementary Figure 4

a

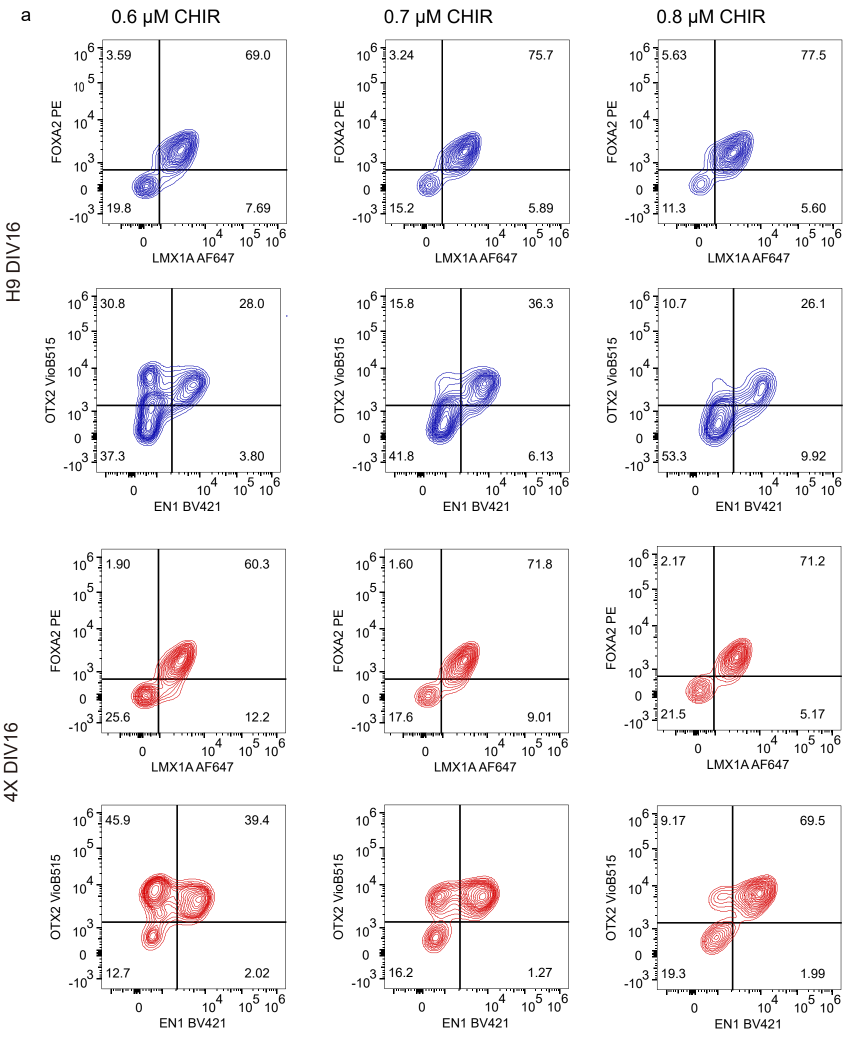


b Gating strategy (applies to all samples):

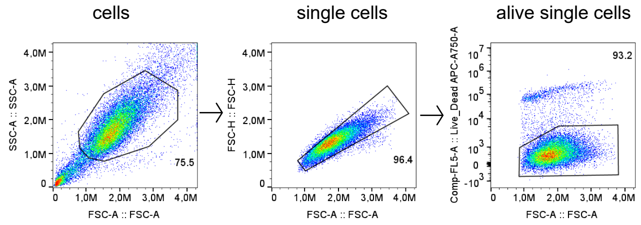


c Confirmed loss of 4X GFP signal after fixation

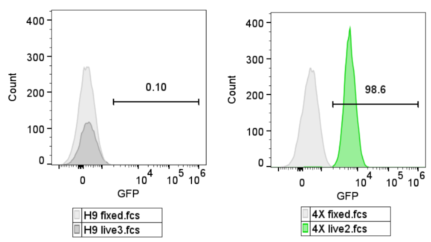


**Supplementary Figure 4: Flow cytometry analysis of FOXA2/LMX1A, OTX2/EN1-positive cells and RNA expression analysis for H9 and 4X at DIV16 midbrain differentiation. a**, Representative flow cytometer analysis plots for FOXA2/LMX1A, OTX2/EN1 for H9 and 4X at DIV16 under different concentrations of GSK3i ranging from 0.6 µM to 0.8 µM. **b**, Gating strategy for flow cytometry analysis. **c,** Confirmed loss of 4X cells' GFP signal after fixation.

Supplementary Figure 5

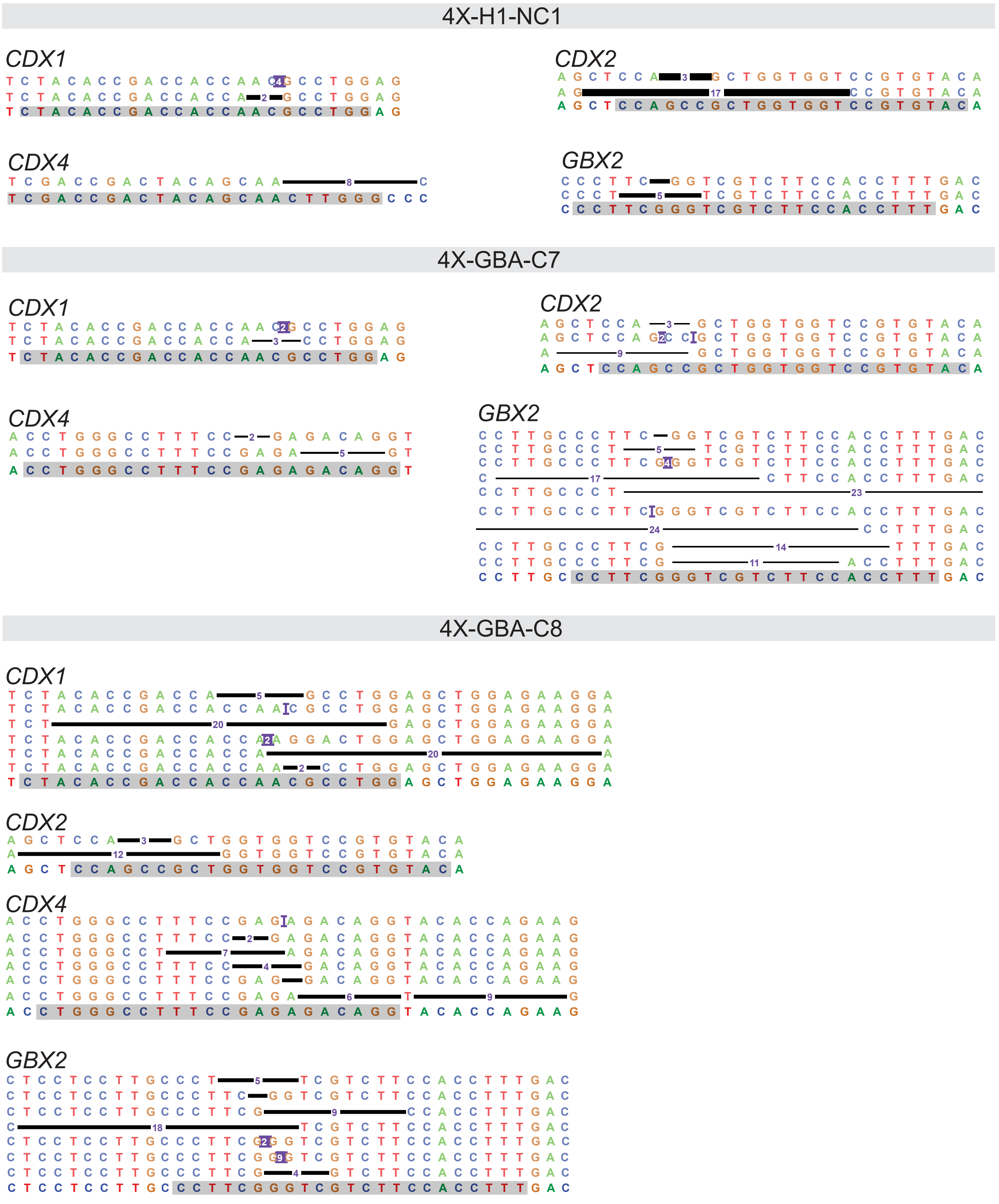


**Supplementary Figure 5: NGS-based amplicon sequencing analysis of *GBX2, CDX1, CDX2* and *CDX4* CRISPR target region of 4X-H1-NC1, 4X-GBA-C7 and 4X-GBA-C8 clones.** 4X-H1-NC1 was identifed as a clonal cell line with knockout of *GBX2* and *CDX1/2/4*. 4X-GBA-C7 and 4X-GBA-C8 were a mixed cell population, both with a knockout of *GBX2* and *CDX1/2/4*. Bottom sequence in each analysis represents the reference sequence, and gray box highlights the CRISPR target sequence.

# Supplementary Figure 6

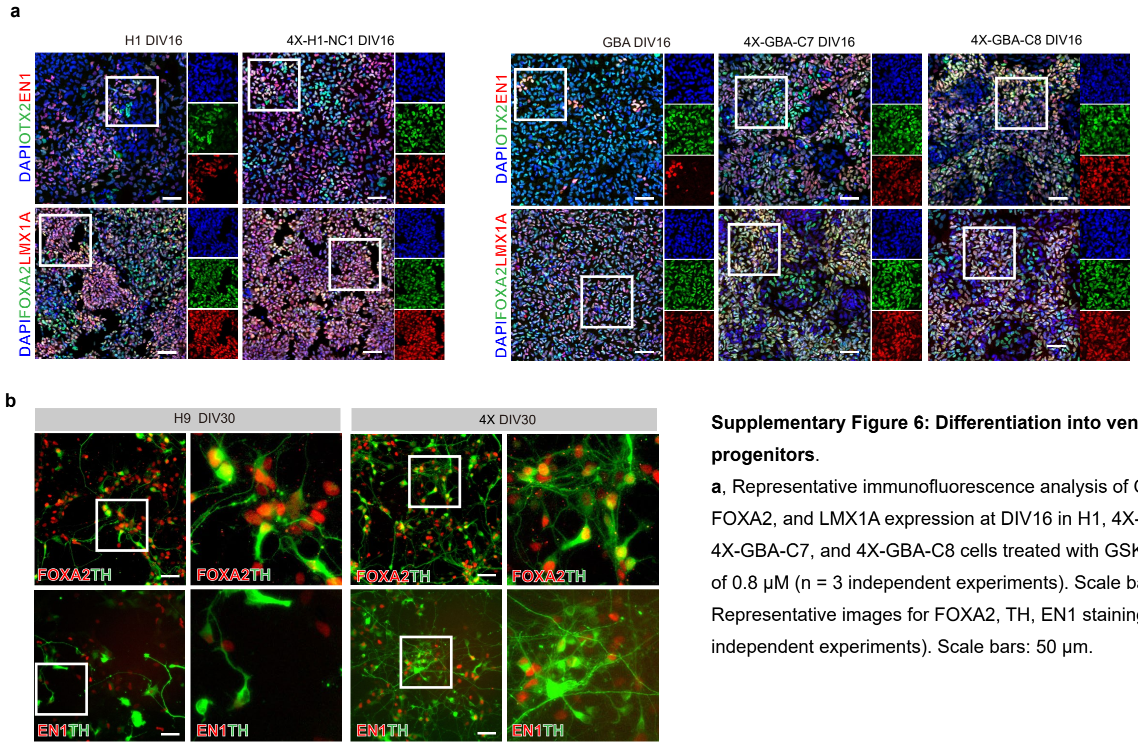


**Supplementary Figure 6: Differentiation into ventral midbrain progenitors**.

**a**, Representative immunofluorescence analysis of OTX2, EN1, FOXA2, and LMX1A expression at DIV16 in H1, 4X-H1-NC1, GBA, 4X-GBA-C7, and 4X-GBA-C8 cells treated with GSK3i at concentration of 0.8 μM (n = 3 independent experiments). Scale bars, 50 μm. **b,** Representative images for FOXA2, TH, EN1 staining at DIV30 (n = 3 independent experiments). Scale bars: 50 μm.

# Supplementary Figure 7

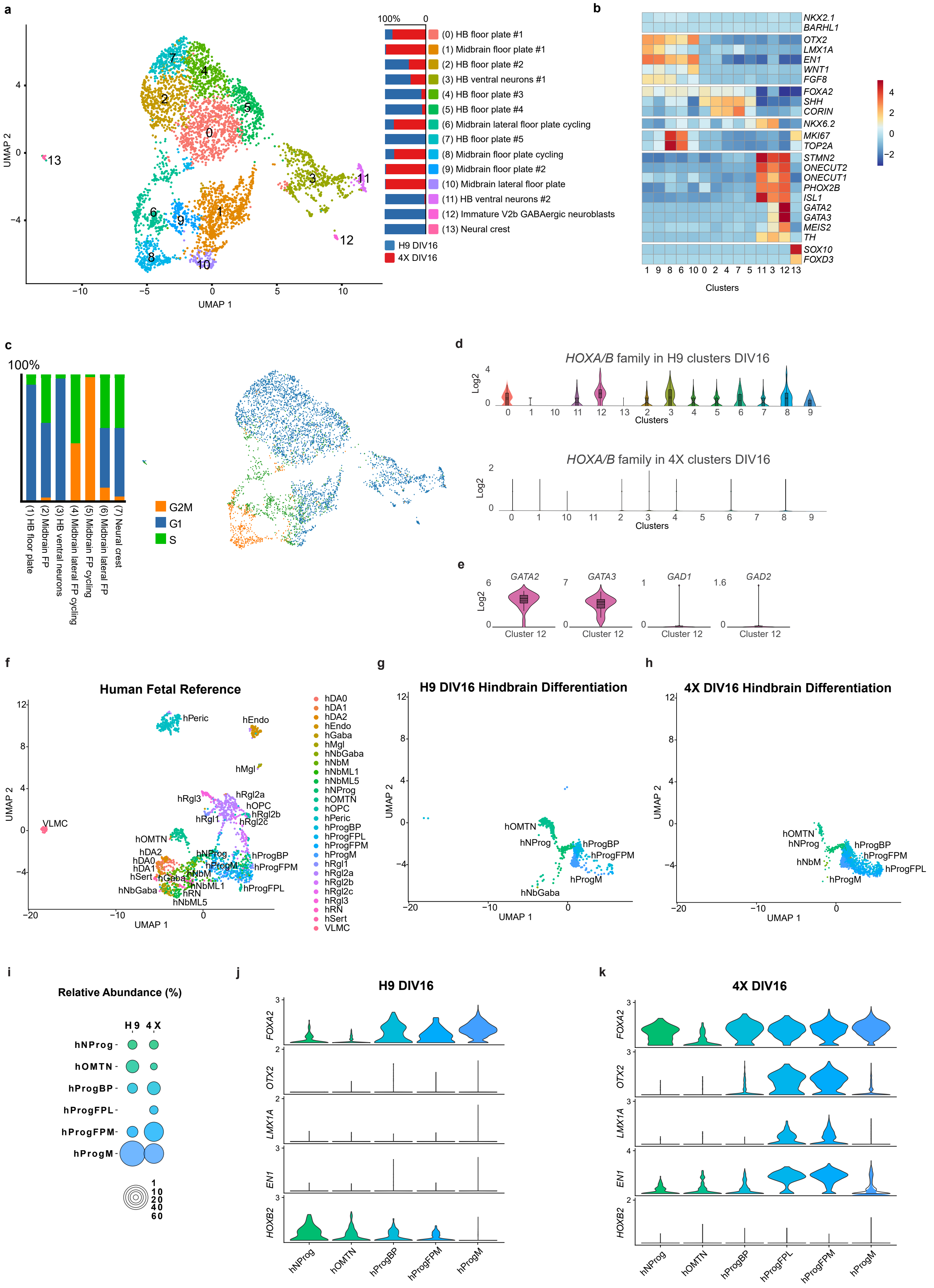


**Supplementary Figure 7: Single-cell sequencing of H9 and 4X cells differentiated under hindbrain condition at DIV16.**
a, UMAP of H9 and 4X cells at DIV16 and a graph of the cluster composition. b, Heatmap of selected genes expressed in each cluster. c, Graph and UMAP of Seurat cell cycle analysis. d, Violin plot of H9 and 4X cells at DIV16 for expression of combined *HOXA/B* family. e, Violin plots of *GATA2*, *GATA3*, *GAD1*, *GAD2* in cluster 12. f, UMAP of human fetal reference data set. g-h, UMAP of H9 (g) and 4X (h) cells at DIV16 after anchoring with reference data. i, Graph showing abundance of H9 and 4X cells in each cluster at DIV16. j-k, Violin plot of H9 (j) and 4X (k) cells at DIV16 for *FOXA2*, *OTX2*, *LMX1A*, *EN1*, *HOXB2* and *HOXA/B*.

## Supplementary Figure 8

**Expression of HOX genes in H9 cell clusters at DIV16 Hindbrain Differentiation**

a

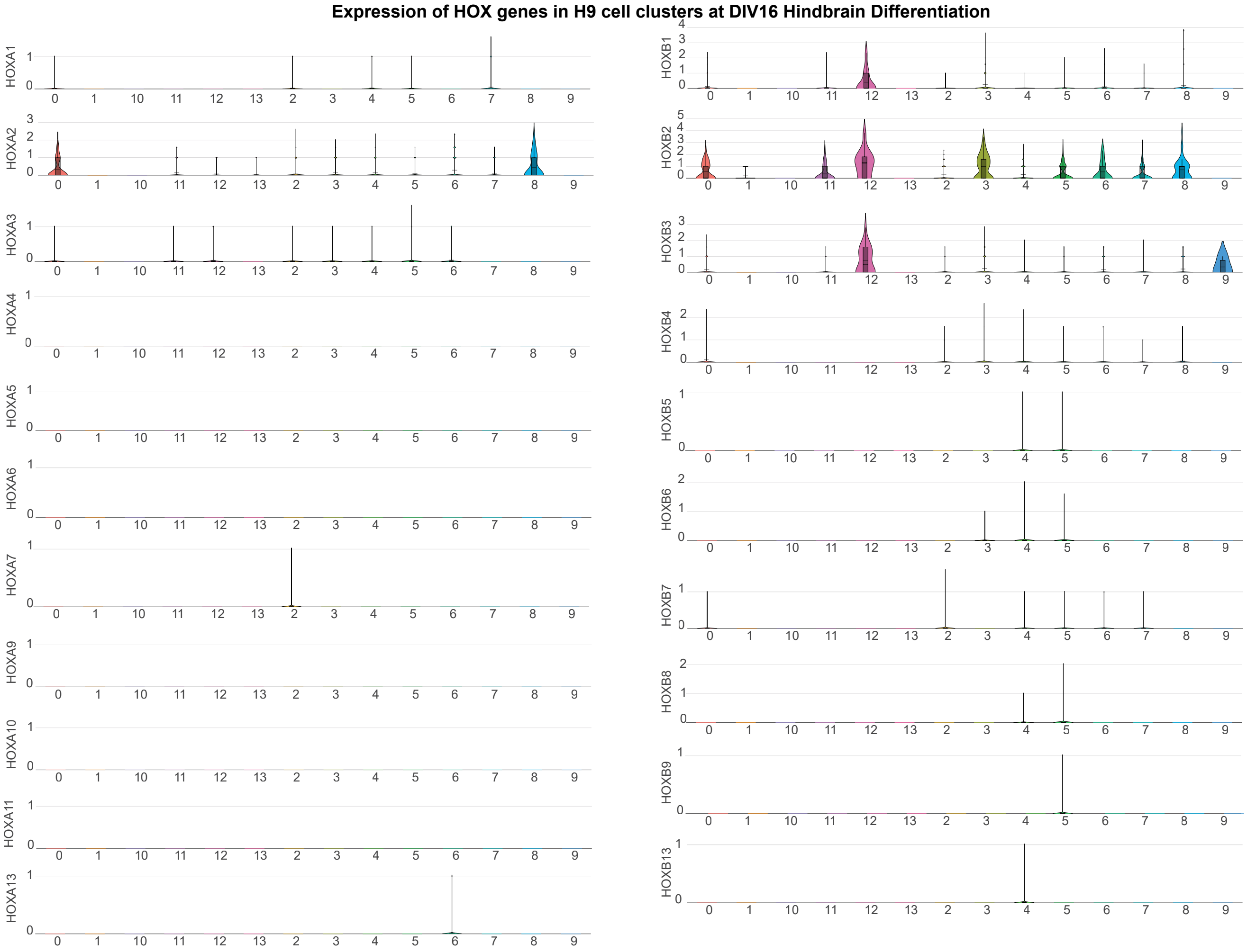


**Expression of HOX genes in 4X cell clusters at DIV16 Hindbrain Differentiation**

b

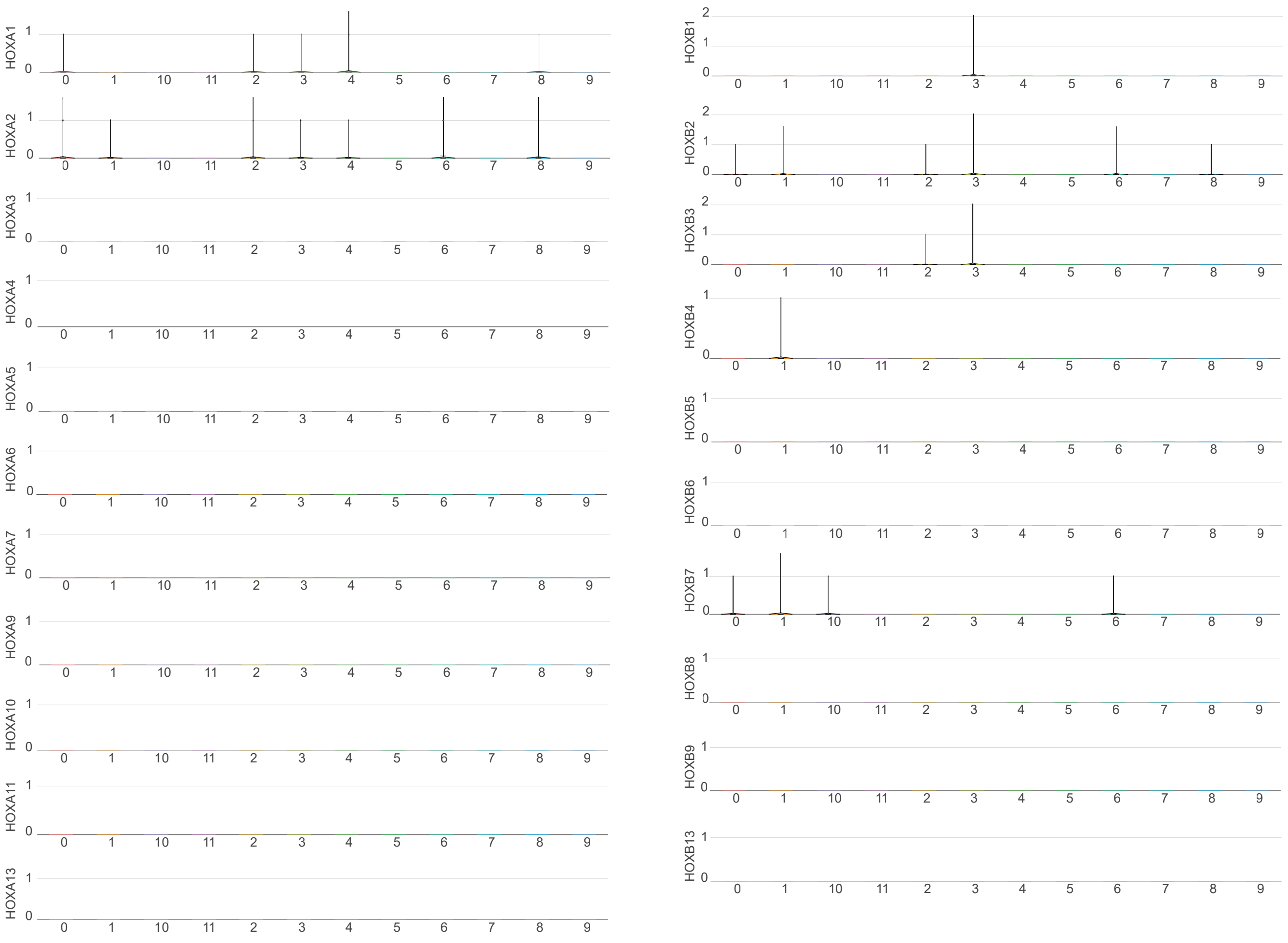


**Supplementary Figure 8: Single-cell sequencing analysis of HOX genes in H9 and 4X cell clusters differentiated under hindbrain condition at DIV16**. a, Expression of HOX genes in H9 cell clusters at DIV16 of hindbrain differentiation. b, Expression of HOX genes in 4X cell clusters at DIV 16 of hindbrain differentiation. Clusters are based on UMAP projection from supplementary Figure 7a.

# Supplementary Figure 9

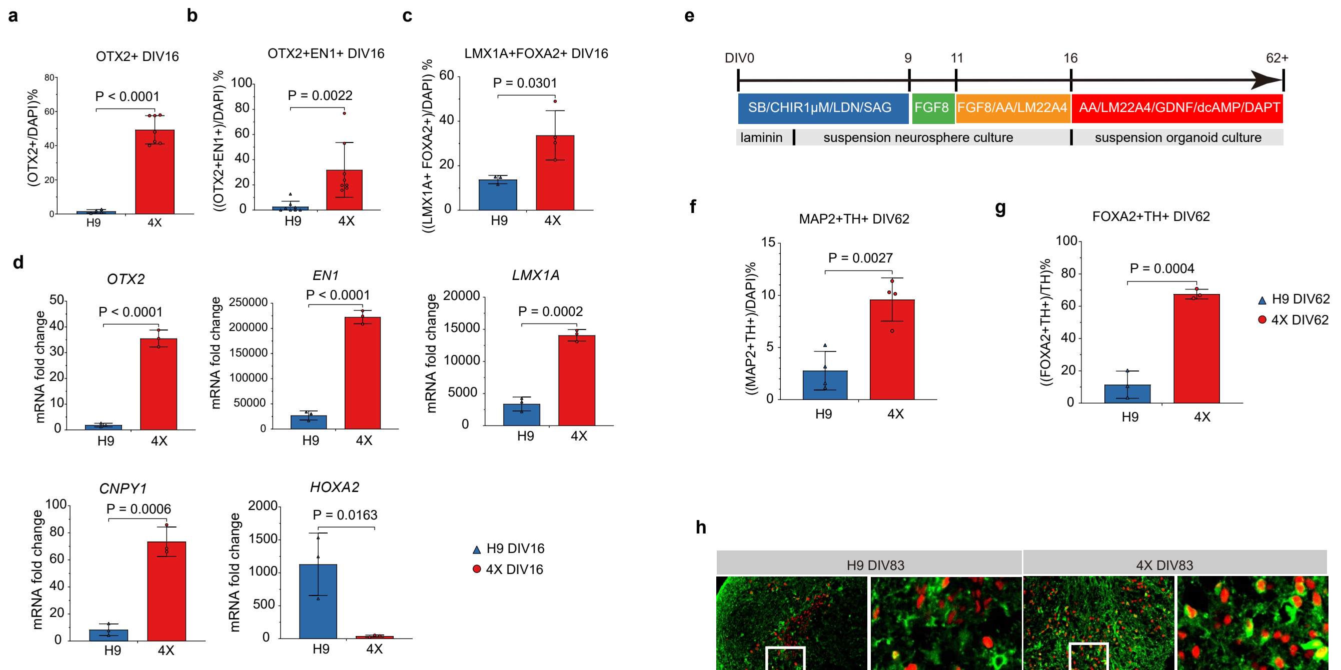


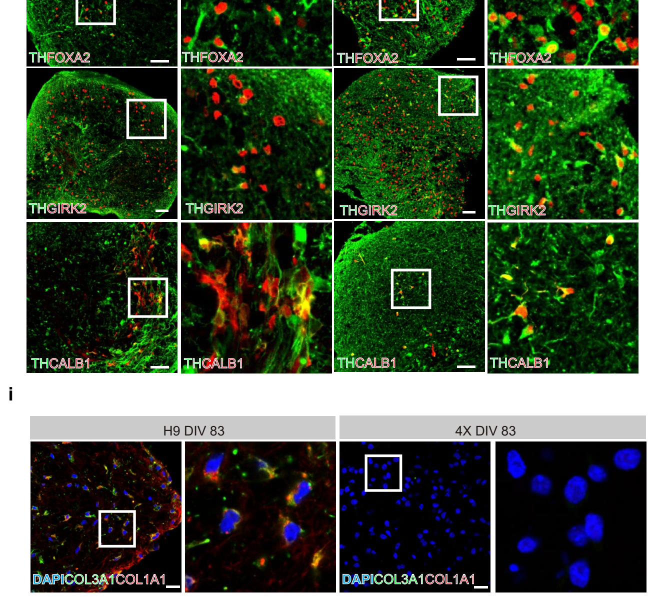


**Supplementary Figure 9: Differentiation under hindbrain condition**.

**a,** Schematic diagram of differentiation protocol under hindbrain condition. **b-d**, Quantification of OTX2,OTX2/EN1,LMX1A/FOXA2 -positive cells among H9 and 4X cells at DIV16 when GSK3i was 1 μM. The data are presented as the mean ± SD; form 2 independent experiments and **(a)** H9 n = 4, 4X n = 7 organoids, for **(b)** n = 8 organoids, for **(c)** H9 n = 3, 4X n = 4 organoids and for **(d)** n = 3 biological replicates. An unpaired two-tailed t-test was used to compare groups. **e**, Expression of *OTX2, EN1, LMX1A, CNPY1*, and *HOXA2* in H9 and 4X cells treated 1 μM of GSK3i at DIV16. The data are presented as the mean ± SD., n = 3. An unpaired two tailed t-test was used to compare groups. **f-g**, Quantification of TH and MAP2 **(f)** and FOXA2/TH **(g)** double positive cells in H9 and 4X cells at DIV62, after treatment with 1 μM of GSK3i. The data are presented as the mean ± SD, form 2 independent experiments and **(f)** n = 4 organoids and for **(g)** n = 3 organoids. An unpaired two-tailed t-test was used to compare groups. **h,** Representative immunofluorescence images of TH/FOXA2, TH/GIRK2, and TH/CALB1 costaining in H9 and 4X cells treated with 1 μM GSK3i on DIV83. Scale bars, 50 μm. **i,** Representative immunofluorescence analysis of COL3A1 and COL1A1 expression at DIV83 (n = 2 independent experiments). Many H9 cells were double positive for COL3A1 and COL1A1, but no 4X cells were positive for COL3A1 or COL1A1. DAPI was used as a nuclear stain. Scale bars, 20 μm.

# Supplementary Figure 10

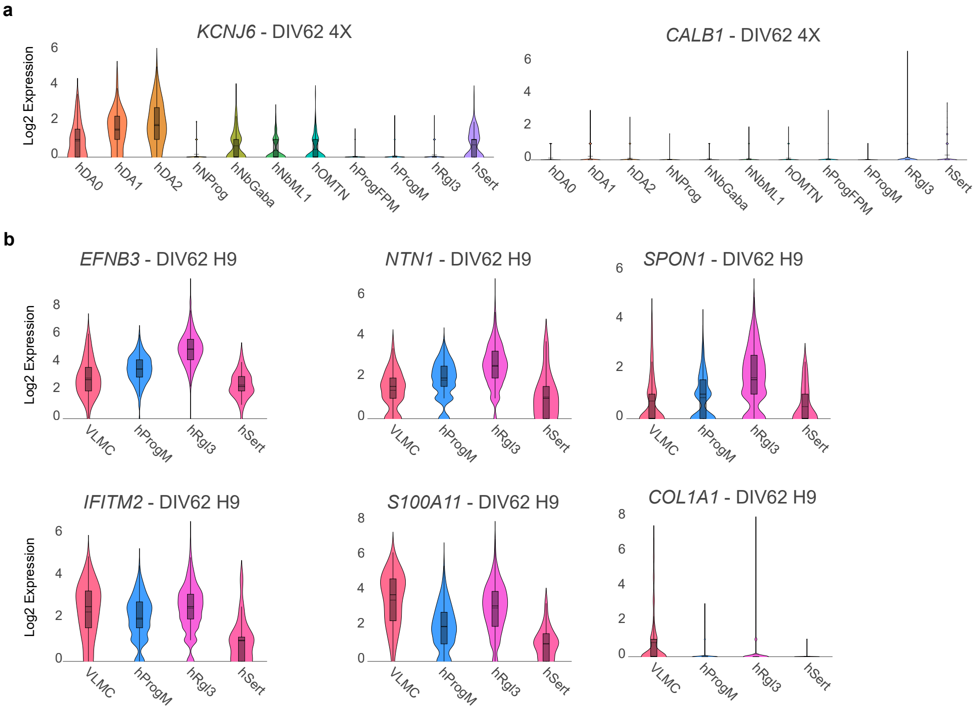


**Supplementary Figure 10: DIV62 Single-Cell Sequencing Data.**
**a**, Violin plot of 4X DIV62 clusters for *KCNJ6 (GIRK2)* and *CALB1*. **b**, Violin plot of H9 DIV62 clusters for *EFNB3*, *NTN1*, *SPON1*, *IFITM2*, *S100A11*, and *COL1A1*.

# Supplementary Figure 11

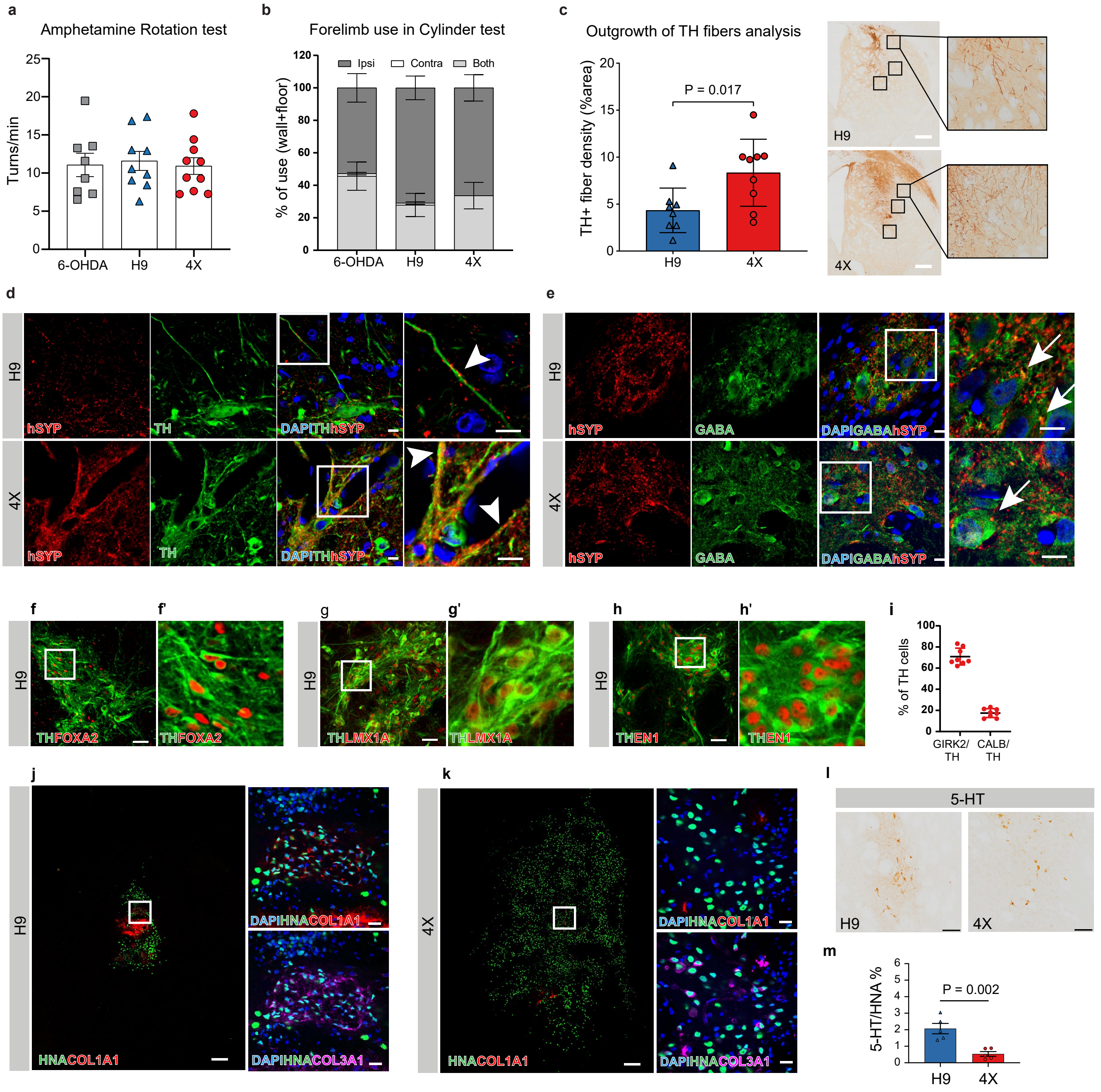


**Supplementary Figure 11: Behavior analysis of 6-OHDA-lesioned rats 3-weeks post lesion and assessment of hindbrain patterned H9 and 4X cell grafts, 18 weeks post-transplantation.**

**a-b**, Amphetamine-induced rotation **(a)** and cylinder tests **(b)** of 6-OHDA-lesioned rats 3-weeks post lesion, showing comparable behavior among 6-OHDA, H9 and 4X groups, subdivided prior to transplantation. For **(a)**, n = 8 for 6-OHDA, n = 9 for H9, n = 10 rats for 4X, mean ± SEM. For **(b)**, n = 7 for 6-OHDA, n = 9 for H9, n = 10 rats for 4X, mean ± SEM. **c,** Outgrowth of TH fibers analysis and representative images. Unpaired two tailed t test was used to compare groups. n = 8 for H9, n = 9 rats for 4X, mean ± SD. Scale bars, 500 µm. **d-e**, Representative immunofluorescence images of H9 and 4X grafts, co-stained with human-specific synaptophysin (hSYP) and TH (d) or GABA (e), n = 9 rats per group. White arrowheads indicate co-localization of hSYP with TH along the TH fibers (d). White arrows indicate colocalization of hSYP with GABA around the GABA neuron cell body (e). Scale bars, 10 µm. **f-h**, Representative immunofluorescence analysis identifies co-staining of TH/FOXA2, TH/LMX1A and TH/EN1 in H9 grafts, 18-weeks post-transplantation (n = 3 rats per group). The squares in **(f-h)** outline the areas of magnification shown on **(f'-h')**. Scale bars, 50 µm. **i,** The percentages of GIRK2/TH and CALB1/TH double-positive cells within TH cells in H9 cell grafts. The data are presented as the mean percentage ± SD (n = 8 rats). **j-k**, Representative photomicrographs and immunofluorescence images of H9 **(i)** and 4X **(j)** grafts, co-stained with HNA/COL1A1 (left and right top panel) and HNA/COL3A1 (right bottom panel), n = 3 rats per group. H9 grafts have many HNA cells positive for COL1A1 and COL3A1, whereas 4X grafts exhibit no or few HNA cells positive for COL1A1 and COL3A1, respectively. DAPI was used as nuclear stain. Scale bars, 200 µm (left panel) and 20 µm (right top and bottom panels). **l**, Representative images of 5-HT cells within H9 and 4X grafts. Scale bars, 100 µm. **m**, Quantification of 5-HT/NHA percentage in H9 and 4X grafts, the data are presented as the mean percentage ± SEM, n = 5 rats, statistic is unpaired two tailed t-test.

# Supplementary Figure 12

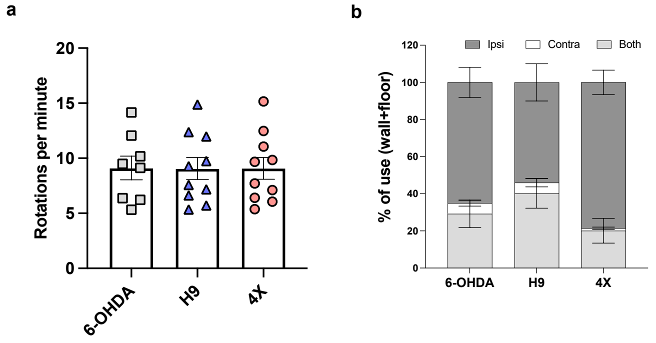


**Supplementary Figure 12: Midbrain patterned in vivo data**

**a-b**, Amphetamine-induced rotation **(a)** and cylinder tests **(b)** of 6-OHDA-lesioned rats 3-weeks post-lesion, showing comparable behavior among 6-OHDA, H9 and 4X groups, subdivided prior to transplantation. For **(a)** n = 8 for 6-OHDA, n = 10 for H9, n = 10 rats for 4X, and for **(b)** n = 5 for 6-OHDA, n = 7 for H9, n = 8 rats for 4X. All data are mean ± SEM.

**Supplementary Table1:**

Guide RNA sequences used to generate knockout cell lines and assessment of indels at exonic off-target sites in *GBX2*$^{-/-}$ and 4X cell line

| Cell lines | Genes | CRISPR target sequence + PAM |
|---|---|---|
| ***GBX2***$^{-/-}$ | *GBX2* | gRNA1: GGCAGCACTACCGGCCGGTA GGG<br>gRNA2: ATGATGATGCAGCGCCCGCT GGG<br>gRNA3: GCATCATCATCAGCGACGGC GGG<br>gRNA4: AAAGGTGGAAGACGACCCGA AGG |
| **4X (*GBX2***$^{-/-}$ ***CDX1/2/4***$^{-/-}$**)**<br>**and**<br>**4X-GBA-C7,**<br>**4X-GBA-C8** | *GBX2*<br>*CDX1*<br>*CDX2*<br>*CDX4* | gRNA4: AAAGGTGGAAGACGACCCGA AGG<br>gRNA1: CTACACCGACCACCAACGCC TGG<br>gRNA1: GTACACGGACCACCAGCGGC TGG<br>gRNA1: CCTGGGCCTTTCCGAGAGAC AGG |
| **4X-H1-NC1** | *GBX2*<br>*CDX1*<br>*CDX2*<br>*CDX4* | gRNA: AAAGGTGGAAGACGACCCGA AGG<br>gRNA: CTACACCGACCACCAACGCC TGG<br>gRNA: GTACACGGACCACCAGCGGC TGG<br>gRNA: TCGACCGACTACAGCAACTT GGG |

***GBX2***$^{-/-}$

| InDels/Seq changes | Locus | Sequence | Mismatch Position | Mismatch Count | cfdOff-target Score | chrom |
|---|---|---|---|---|---|---|
| **YES** | **GBX2** | **ATGATGATGCAGCGCCCGCTGGG** | ……………….. | 0 | 1,000 | **Chr2** |
| No indels | VAX1 | GCGGGGATGCAGCGCCCGCTGGG | **.**............... | 4 | 0,343 | chr10 |
| No indels | FBN2 | ATGAGGACGCAGCGCACCCTGGG | ....*..*.......*.*.. | 4 | 0,305 | chr5 |
| No indels | BCL9L | ATGGGCATGCAGCGCCCCCTGGG | ...***...........*.. | 4 | 0,130 | chr11 |
| No indels | STOX2 | ATGATGAAGCAGCCCTCCCTGGG | .......*.....*.*.*.. | 4 | 0,050 | chr4 |
| No indels | TNS3 | ATGACGATGCAGATCCTGCTGGG | ....*.......**..*... | 4 | 0,048 | chr7 |
| No indels | LRP1 | ATGGGGATGCAGCGCCCACTAGG | ...**............*.. | 3 | 0,277 | chr12 |
| **YES** | **GBX2** | **GGCAGCACTACCGGCCGGTAGGG** | ……………….. | 0 | 1,000 | **Chr2** |
| No indels | ZNF26 | AGCAGCACTACCTGCAGGGAGGG | *...........*..*..*. | 4 | 0,068 | chr12 |
| No indels | DDX12P | GGCAGCACCACCAGCTGGGAGGG | ........*...*..*..*. | 4 | 0,066 | chr12 |
| No indels | RP11-22B23.1 | GGCAGCACCACCAGCTGGGAGGG | ........*...*..*..*. | 4 | 0,066 | chr12 |
| No indels | DDX11 | GGCAGCACCACCAGCTGGGAGGG | ........*...*..*..*. | 4 | 0,066 | chr12 |
| No indels | RNA5SP18 | GGCTGCACTACCGGCGTCTAAGG | ...*...........***.. | 4 | 0,012 | chr1 |
| No indels | LIPG | TCCAGCAATCCCGGCCGGTAAGA | **.....*.*.......... | 4 | 0,010 | chr18 |
| No indels | B4GALT7 | AGCAGCACTACCGGCTGGTGAGG | *.............*...* | 3 | 0,212 | chr5 |
| No indels | TMEM150C | GGCAGCAGTAGCGGCGGGTAGGG | .......*..*....*.... | 3 | 0,025 | chr4 |
| No indels | GBX1 | GGCAGCACGAGCGGCCGGTAGGG | ........*.*......... | 2 | 0,155 | chr7 |
| **YES** | **GBX2** | **GCATCATCATCAGCGACGGCGGG** | ……………….. | 0 | 1,000 | **Chr2** |
| No indels | PDPN | TCGTCCCCATCAGCGACGGCAGG | *.*..**............. | 4 | 0,191 | chr1 |
| No indels | ARFGAP1 | GGAACAGCAACAGCGACGGCGGG | .*.*..*..*.......... | 4 | 0,167 | chr20 |
| No indels | KALRN | GTTACATCACCAGCGACGGCTGG | .***.....*.......... | 4 | 0,163 | chr3 |
| No indels | SLC16A8 | CCAGCATGATGAGCGACGGCTGG | *..*...*..*......... | 4 | 0,072 | chr22 |
| No indels | IER2 | GCAGCAGCCTGAGCGACGGCGGG | ...*..*.*.*......... | 4 | 0,053 | chr19 |
| No indels | HMCN2 | GCATCATCCCCACAGACGGCGGG | ........**..**...... | 4 | 0,045 | chr9 |
| No indels | ZFC3H1 | GCAGCAGCAGCAGCGGCGGCGGG | ...*..*..*.....*.... | 4 | 0,035 | chr12 |
| No indels | OLFM2 | GCAGCAGCAGCAGCGGCGGCGGG | ...*..*..*.....*.... | 4 | 0,035 | chr19 |
| No indels | CASZ1 | GCCTCATCACCAGCGGCCGCAGG | ..*......*.....*.*.. | 4 | 0,021 | chr1 |

| | | | | | | |
|---|---|---|---|---|---|---|
| No indels | PARD3 | TCATCATCATCATCTACCGCGGG | *...........*.*..*.. | 4 | 0,020 | chr10 |
| No indels | MYO1C | GCATCAACATCAACCAGGGCAGG | ......*.....*.*.*... | 4 | 0,013 | chr17 |
| No indels | UNC45B | GAATCATGATCAGCTGCGGCAGG | .*.....*......**.... | 4 | 0,013 | chr17 |
| **YES** | **GBX2** | **AAAGGTGGAAGACGACCCGAAGG** | ……………….. | 0 | 1,000 | **Chr2** |
| No indels | TBX2 | CGAGGTGGAGGACGACCCCAAGG | **.......*........*. | 4 | 0,171 | chr17 |
| No indels | XPO7 | AAAGGTGGAAAACTACCCTGAGG | ..........*..*....** | 4 | 0,136 | chr8 |
| No indels | DDX10P2 | AAAGGTGGAAAAAGACTCAAAGG | ..........*.*...*.*. | 4 | 0,128 | chr9 |
| No indels | GPR25 | CCAGGTGGAAGACGGCCCGCAGG | **............*....* | 4 | 0,099 | chr1 |
| No indels | TEX13A | GAAGGTGGAAGACAAGCTGAGGG | *............*.*.*.. | 4 | 0,074 | chrX |
| No indels | INCA1 | GAGGGTGGATGAGGACCCGAGGG | *.*......*..*....... | 4 | 0,074 | chr17 |
| No indels | RP11-381P6.1 | AAAGGTGGAGGACATCCCTATGG | .........*...**...*. | 4 | 0,056 | chr17 |
| No indels | IRF2BPL | AGAGGTCGAAGACGACGCGGAGG | .*....*.........*..* | 4 | 0,025 | chr14 |
| No indels | AL359195.1 | AAAGGTGGATGAGGTCCCGGAGG | .........*..*.*....* | 4 | 0,018 | chr10 |
| No indels | RPL5P5 | AAATGTAGAAGACGACGAGAAGG | ...*..*.........**.. | 4 | 0,012 | chr1 |
| No indels | STARD6 | AAAGCTGGATGATGACCCGAAGG | ....*....*..*....... | 3 | 0,485 | chr18 |
| No indels | TBX3 | AGAGGTGGAGGACGACCCCAAGG | .*.......*........*. | 3 | 0,199 | chr12 |
| No indels | DDX10 | AAAGGTGGAAAAAGACTCGAAGG | ..........*.*...*... | 3 | 0,179 | chr11 |

# 4X (*GBX2*$^{-/-}$ *CDX1/2/4*$^{-/-}$)

***CDX1***

| InDels/ Seq changes | Locus | Sequence | Mismatch Position | Mismatch Count | CFD Off-target Score | chrom |
|---|---|---|---|---|---|---|
| **YES** | ***CDX1*** | **CTACACCGACCACCAACGCCTGG** | ……………….. | 0 | 1,000 | **chr5** |
| No indels | *LRP2BP* | GAACACCAACCACCAACTCCAGG | **.....*.........*.. | 4 | 0,515 | chr4 |
| No indels | *ATP6AP2* | CCTCACCCGCCACCAACGCCAGG | .**....**........... | 4 | 0,248 | chrX |
| No indels | *FAM53A* | GGACTCTGACCACCAACGCCCGG | **..*.*............. | 4 | 0,227 | chr4 |
| No indels | *GUSBP11* | CTTCTCCGACAACCGACGCCGGG | ..*.*.....*...*..... | 4 | 0,125 | chr22 |
| No indels | *NMD3* | CCACGCCGACCCCCAACCCCGGG | .*..*......*.....*.. | 4 | 0,077 | chr3 |
| No indels | *DLX2* | CTACACCAACCAGCAGCACCCGG | .......*....*..*.*.. | 4 | 0,018 | chr2 |
| No indels | *CDX2* | GTACACGGACCACCAGCGGCTGG | *.....*........*..*. | 4 | 0,010 | chr13 |
| No indels | *HSPA1B* | CTACTCCGACAACCAACCCGGGG | ....*.....*......*.* | 4 | 0,008 | chr6 |
| No indels | *HSPA1B* | CTACTCCGACAACCAACCCGGGG | ....*.....*......*.* | 4 | 0,008 | chr6 |
| No indels | *HSPA1A* | CTACTCCGACAACCAACCCGGGG | ....*.....*......*.* | 4 | 0,008 | chr6 |
| No indels | *HSPA1B* | CTACTCCGACAACCAACCCGGGG | ....*.....*......*.* | 4 | 0,008 | chr6 |
| No indels | *HSPA1A* | CTACTCCGACAACCAACCCGGGG | ....*.....*......*.* | 4 | 0,008 | chr6 |
| No indels | *HSPA1A* | CTACTCCGACAACCAACCCGGGG | ....*.....*......*.* | 4 | 0,008 | chr6 |
| No indels | *HSPA1B* | CTACTCCGACAACCAACCCGGGG | ....*.....*......*.* | 4 | 0,008 | chr6 |
| No indels | *HSPA1A* | CTACTCCGACAACCAACCCGGGG | ....*.....*......*.* | 4 | 0,008 | chr6 |
| No indels | *HSPA1A* | CTACTCCGACAACCAACCCGGGG | ....*.....*......*.* | 4 | 0,008 | chr6 |
| No indels | *HSPA1B* | CTACTCCGACAACCAACCCGGGG | ....*.....*......*.* | 4 | 0,008 | chr6 |
| No indels | *EFCAB4A* | CCTCACCGACCACGCACGCCGGG | .**..........**..... | 4 | 0,000 | chr11 |
| No indels | *CATSPERG* | CTACACCGCCCCCCAACCCCTGG | ........*..*.....*.. | 3 | 0,072 | chr19 |

***CDX2***

| InDels/ Seq changes | Locus | Sequence | Mismatch Position | Mismatch Count | CFD Off target Score | chrom |
|---|---|---|---|---|---|---|
| **YES** | ***CDX2*** | **GTACACGGACCACCAGCGGCTGG** | ……………….. | 0 | 1,000 | **ch13** |

| | | | | | | |
|---|---|---|---|---|---|---|
| No indels | *CDX1* | CTACACCGACCACCAACGCCTGG | *.....*........*..*. | 4 | 0,220 | chr5 |
| No indels | *KPTN* | GGACACCCCCCACCAGCGGCTGG | .*....***.......... | 4 | 0,203 | chr19 |
| No indels | *MYH10* | GGACCTGGACCACCAGCGCCAGG | .*..**............*. | 4 | 0,175 | chr17 |
| No indels | *CSPG5* | GAAGCCGGCCCACCAGCGGCTGG | .*.**...*.......... | 4 | 0,121 | chr3 |
| No indels | *SEPT9* | GCACGCGGACCTCCAGTGGCCGG | .*..*......*....*... | 4 | 0,096 | chr17 |
| No indels | *HEATR2* | GAACCCGGAGCACCAGCCGCTGG | .*..*....*.......*.. | 4 | 0,078 | chr7 |
| No indels | *INTS10* | GGACACGCACCAGCAGCAGCGGG | .*.....*....*....*.. | 4 | 0,049 | chr8 |
| No indels | *FER1L5* | GTTCAGGGACCCCCAGAGGCAGG | ..*..*.....*....*... | 4 | 0,043 | chr2 |
| No indels | *VIT* | GTACACCTACGAACAGCGGCTGG | ......**..*.*....... | 4 | 0,041 | chr2 |
| No indels | *ANKRD13B* | GTACCGGGACTACCAGCGGGTGG | ....**....*........* | 4 | 0,005 | chr17 |
| No indels | *EFCAB4A* | GTACACGGAGGGCCAGGGGCTGG | .........***....*... | 4 | 0,004 | chr11 |
| No indels | *HPCAL1* | GTTCACCGACCACGAGCTGCAGG | ..*...*......*...*.. | 4 | 0,000 | chr2 |
| No indels | *TFCP2L1* | GTATACGGAGCACCAGCAGCTGG | ...*.....*.......*.. | 3 | 0,215 | chr2 |
| No indels | *MRPL28* | GTCCTGGGACCACCAGCGGCTAG | ..*.**............. | 3 | 0,020 | chr16 |

***CDX4***

| InDels/ Seq changes | Off target locus | off target Seq | mismatchPos | Mismatch Count | CFD Off target Score | chrom |
|---|---|---|---|---|---|---|
| **YES** | ***CDX4*** | **CCTGGGCCTTTCCGAGAGAC AGG** | **…………………** | **0** | **1,000** | **chrX** |
| No indels | *HOXD8* | CCTAGCCCTCACCGAGAGACAGG | ...*.*...**......... | 4 | 0,245 | chr2 |
| No indels | *FAM86C1* | CCTCAGCCTTGCCGGGAGACCGG | ...**.....*...*..... | 4 | 0,119 | chr11 |
| No indels | *HOXB8* | CCTGGGACTGACAGAGAGACAGG | ......*..**.*....... | 4 | 0,108 | chr17 |
| No indels | *C1orf127* | CCTGGGGCTCTCCGGGAGAACGG | ......*..*....*....* | 4 | 0,082 | chr1 |
| No indels | *SZRD1/SPATA21* | TCTGGGCCTTGCCTGGAGACTGG | *.........*..**..... | 4 | 0,069 | chr1 |
| No indels | *RP11-512N21.1* | CCTGTTCCTTTCTCAGAGACAGG | ....**......**...... | 4 | 0,060 | chr12 |
| No indels | *C3orf22* | CAGGGGCCTCTCCTAGAGACTGG | .**......*...*...... | 4 | 0,052 | chr3 |
| No indels | *SCN8A* | CCTGGGCGGTTCCGAGACCCGGG | .......**........**. | 4 | 0,039 | chr12 |
| No indels | *RP11-798K23.3* | CCTGGGCCTCTCCTGGAGGCAGG | .........*...**...*. | 4 | 0,035 | chr5 |
| No indels | *AC136604.1* | CCTGGGCCTCTCCTGGAGGCAGG | .........*...**...*. | 4 | 0,035 | chr5 |
| No indels | *C5orf60* | CCTGGGCCTCTCCTGGAGGCAGG | .........*...**...*. | 4 | 0,035 | chr5 |
| No indels | *GPBAR1* | CCTGGGCCTTCCTGAGTGTCAGG | ..........*.*...*.*. | 4 | 0,034 | chr2 |
| No indels | *UBB* | GCTGGGCCTTTCCGGGACAGTGG | *.............*..*.* | 4 | 0,017 | chr17 |
| No indels | *RP11-313A24.1* | GCTGGGCCGTTCCCAGAGAGAGG | *.......*....*.....* | 4 | 0,014 | chr1 |
| No indels | *C9orf89* | CCTGGGCCTTGCTGTGGGACTGG | ..........*.*.*.*... | 4 | 0,010 | chr9 |
| No indels | *EBF1* | CCTGGCCCTCTACGACAGACAGG | .....*...*.*...*.... | 4 | 0,000 | chr5 |

***GBX2***

| InDels/ Seq changes | Off target locus | Sequence | Mismatch Position | Mismatch Count | CFD Off target Score | chrom |
|---|---|---|---|---|---|---|
| **YES** | ***GBX2*** | **AAAGGTGGAAGACGACCCGAAGG** | **…………………** | **0** | **1,000** | **Chr2** |
| No indels | *TBX2* | CGAGGTGGAGGACGACCCCAAGG | **.......*........*. | 4 | 0,171 | chr17 |
| No indels | *XPO7* | AAAGGTGGAAAACTACCCTGAGG | ..........*..*....** | 4 | 0,136 | chr8 |
| No indels | *DDX10P2* | AAAGGTGGAAAAAGACTCAAAGG | ..........*.*...*.*. | 4 | 0,128 | chr9 |
| No indels | *GPR25* | CCAGGTGGAAGACGGCCCGCAGG | **............*....* | 4 | 0,099 | chr1 |
| No indels | *TEX13A* | GAAGGTGGAAGACAAGCTGAGGG | *............*.*.*.. | 4 | 0,074 | chrX |
| No indels | *INCA1* | GAGGGTGGATGAGGACCCGAGGG | *.*......*..*....... | 4 | 0,074 | chr17 |
| No indels | *RP11-381P6.1* | AAAGGTGGAGGACATCCCTATGG | .........*...**...*. | 4 | 0,056 | chr17 |

| | | | | | | |
|---|---|---|---|---|---|---|
| No indels | *IRF2BPL* | AGAGGTCGAAGACGACGCGGAGG | .*....*.........*..* | 4 | 0,025 | chr14 |
| No indels | *AL359195.1* | AAAGGTGGATGAGGTCCCGGAGG | .........*..*.*....* | 4 | 0,018 | chr10 |
| No indels | *RPL5P5* | AAATGTAGAAGACGACGAGAAGG | ...*..*.........**.. | 4 | 0,012 | chr1 |
| No indels | *STARD6* | AAAGCTGGATGATGACCCGAAGG | ....*....*..*....... | 3 | 0,485 | chr18 |
| No indels | *TBX3* | AGAGGTGGAGGACGACCCCAAGG | .*.......*........*. | 3 | 0,199 | chr12 |
| No indels | *DDX10* | AAAGGTGGAAAAAGACTCGAAGG | ..........*.*...*... | 3 | 0,179 | chr11 |

**Supplementary Table 2:**
Taqman QPCR primers.

| Genes | Assay ID |
|---|---|
| *OTX2* | Hs00222238_m1 |
| *LMX1A* | Hs00898455_m1 |
| *CDX2* | Hs01078080_m1 |
| *MAFB* | Hs00534343_s1 |
| *HOXA2* | Hs00534579_m1 |
| *HOXA3* | Hs00601076_m1 |
| *HOXB1* | Hs00157973_m1 |
| *HOXB2* | Hs01911167_s1 |
| *CNPY1* | Hs01073160_m1 |
| *BARHL1* | Hs01063929_m1 |
| *IRX3* | Hs01124217_g1 |
| *RPL32* | Hs00851655_g1 |

**Supplementary Table 3:**

Nanostring probes.

| Genes | Probe sequence |
|---|---|
| *OTX2* | GGCTGGACATTCCAGTTTTAGCCAGGCATTGGTTAAAAGAGTTAGATGGGATGATGCTCAGACTCATCTGATCAAAGTTCCGAGAGGCATAGAAGGAAAA |
| *EN1* | GCAGCATTTTTGAAAAGGGAGAAAGACTCGGACAGGTGCTATCGAAAAATAAGATCCATTCTCTATTCCCAGTATAAGGGACGAAACTGCGAACTCCTTA |
| *PAX8* | GACACTGTGCCCAGTGTCAGCTCCATTAATAGAATCATCCGGACCAAAGTGCAGCAACCATTCAACCTCCCTATGGACAGCTGCGTGGCCACCAAGTCCC |
| *HOXA2* | CCCAAAGTTTCCCAGTCTCGCCTTTAACCAGCAATGAGAAAAATCTGAAACATTTTCAGCACCAGTCACCCACTGTTCCCAACTGCTTGTCAACAATGGG |
| *HOXA4* | TGCACTTCACAAATTAATGACCATGAGCTCGTTTTTGATAAACTCCAACTACATCGAGCCCAAGTTCCCTCCCTTCGAGGAGTACGCGCAGCACAGCGGC |
| *HOXB8* | GTGGTAGTATCTCGTAATAGCTTCTGTGTGTGAGCTACCGTGGATCTCCTTCCCTTCTCTTGGGGGCCGGGGGGAAAGAAAAGGATTTAAGCAAAGGCTC |
| *HOXC10* | GGAAAGTTCGGCTAGTGTTCGTGTGTTTGTCGTAGCACCCAGAGCCTCCACCAAACCCTCTCCATGTCTTTACCTCCCAGTCGCTCTAAGAATCTGCTTG |
| *PAX2* | TCCTCCTCCGGCAGGAACTGAACAGAACCACAAAAAGTCTACATTTATTTAATATGATGGTCTTTGCAAAAAGGAACAAAACAACACAAAAGCCCACCAG |
| *PAX5* | CTCCAAGAGGAGCACACTTTGGGGAGATGTCCTGGTTTCCTGCCTCCATTTCTCTGGGACCGATGCAGTATCAGCAGCTCTTTTCCAGATCAAAGAACTC |
| *FGF8* | AGAGCAACGGCAAAGGCAAGGACTGCGTCTTCACGGAGATTGTGCTGGAGAACAACTACACAGCGCTGCAGAATGCCAAGTACGAGGGCTGGTACATGGC |
| *IRX3* | AGTCGCTTCTGTGGCACCCCGCATTCGCTGTGAGGTTTGTTTGTCCGGTTGATTTTGGGGGGTGGAGTTTCAGTGAGAATAAACGTGTCTGCCTTTGTGT |
| *HOXA1* | CAGATAATTCTGGACCAGAGACTTGGTGCGGGGTTAACACCTTCATCCAGATTGGGTGCCAGCATACATTTTCTGGTGGGCCTTAACATCCCTCCTGCTT |
| *HOXC6* | ACGTCGCCCTCAATTCCACCGCCTATGATCCAGTGAGGCATTTCTCGACCTATGGAGCGGCCGTTGCCCAGAACCGGATCTACTCGACTCCCTTTTATTC |
