# Supplementary material for: Enhanced production of mesencephalic dopaminergic neurons from lineage-restricted human undifferentiated stem cells
Source: Nat Commun. 2023 Dec 5;14:7871. doi: 10.1038/s41467-023-43471-0 (PMC10698156; doi:10.1038/s41467-023-43471-0)
Supplement: Supplementary file 3 — Reporting Summary [file 41467_2023_43471_MOESM3_ESM.pdf]

nature portfolio

Corresponding author(s): Mark Denham

Last updated by author(s): 1 Nov 2023

Reporting Summary

Nature Portfolio wishes to improve the reproducibility of the work that we publish. This form provides structure for consistency and transparency in reporting. For further information on Nature Portfolio policies, see our Editorial Policies and the Editorial Policy Checklist.

Statistics

For all statistical analyses, confirm that the following items are present in the figure legend, table legend, main text, or Methods section.

n/a	Confirmed	
☐	☒	The exact sample size (*n*) for each experimental group/condition, given as a discrete number and unit of measurement
☐	☒	A statement on whether measurements were taken from distinct samples or whether the same sample was measured repeatedly
☐	☒	The statistical test(s) used AND whether they are one- or two-sided<br>*Only common tests should be described solely by name; describe more complex techniques in the Methods section.*
☒	☐	A description of all covariates tested
☐	☒	A description of any assumptions or corrections, such as tests of normality and adjustment for multiple comparisons
☐	☒	A full description of the statistical parameters including central tendency (e.g. means) or other basic estimates (e.g. regression coefficient) AND variation (e.g. standard deviation) or associated estimates of uncertainty (e.g. confidence intervals)
☐	☒	For null hypothesis testing, the test statistic (e.g. *F*, *t*, *r*) with confidence intervals, effect sizes, degrees of freedom and *P* value noted<br>*Give P values as exact values whenever suitable.*
☒	☐	For Bayesian analysis, information on the choice of priors and Markov chain Monte Carlo settings
☒	☐	For hierarchical and complex designs, identification of the appropriate level for tests and full reporting of outcomes
☒	☐	Estimates of effect sizes (e.g. Cohen's *d*, Pearson's *r*), indicating how they were calculated

Our web collection on statistics for biologists contains articles on many of the points above.

Software and code

Policy information about availability of computer code

Data collection	Cell Ranger Single-Cell Software Suite (v. 3.1.0); Zen Software (Zeiss Zen Black); NovoCyte Quanteon analyzer (Acea Biosciences Inc., Santa Clara, CA); CytoFLEX S flow cytometer (Beckman Coulter, Indianapolis, IN, USA)
Data analysis	Zen Software (ZEN 3.3 blue edition); FlowJo software (v10.7.2, Ashland, OR, USA); CytExpert software; ImageJ software (v.1.53); Fiji software (v1.0);QuPath software(v.0.2.3); ; FeatureCount (v1.6.4); edgeR (v3.32); Seurat R package (v 3.2.1); R package DoubletFinder (v.2.0.3); Loupe Browser 5(v.5.1); Clampex 10.6 software; Clampfit (v.10.6); GraphPad Prism (v 9.1.1.225)

For manuscripts utilizing custom algorithms or software that are central to the research but not yet described in published literature, software must be made available to editors and reviewers. We strongly encourage code deposition in a community repository (e.g. GitHub). See the Nature Portfolio guidelines for submitting code & software for further information.

Data

Policy information about availability of data

All manuscripts must include a data availability statement. This statement should provide the following information, where applicable:

- Accession codes, unique identifiers, or web links for publicly available datasets
- A description of any restrictions on data availability
- For clinical datasets or third party data, please ensure that the statement adheres to our policy

The data generated in this study is available in the source data file.

RNA-seq data generated from this study have been deposited in NCBI's Gene Expression Omnibus with the accession number GEO: GSE227071
https://www.ncbi.nlm.nih.gov/geo/query/acc.cgi?acc=GSE227071
The Human genome build hg38 (Ensembl release 92) is available at https://www.ensembl.org/Homo_sapiens/Info/Index

Research involving human participants, their data, or biological material

Policy information about studies with human participants or human data. See also policy information about sex, gender (identity/presentation), and sexual orientation and race, ethnicity and racism.

Reporting on sex and gender	*Use the terms sex (biological attribute) and gender (shaped by social and cultural circumstances) carefully in order to avoid confusing both terms. Indicate if findings apply to only one sex or gender; describe whether sex and gender were considered in study design; whether sex and/or gender was determined based on self-reporting or assigned and methods used. Provide in the source data disaggregated sex and gender data, where this information has been collected, and if consent has been obtained for sharing of individual-level data; provide overall numbers in this Reporting Summary. Please state if this information has not been collected. Report sex- and gender-based analyses where performed, justify reasons for lack of sex- and gender-based analysis.*
Reporting on race, ethnicity, or other socially relevant groupings	*Please specify the socially constructed or socially relevant categorization variable(s) used in your manuscript and explain why they were used. Please note that such variables should not be used as proxies for other socially constructed/relevant variables (for example, race or ethnicity should not be used as a proxy for socioeconomic status). Provide clear definitions of the relevant terms used, how they were provided (by the participants/respondents, the researchers, or third parties), and the method(s) used to classify people into the different categories (e.g. self-report, census or administrative data, social media data, etc.) Please provide details about how you controlled for confounding variables in your analyses.*
Population characteristics	*Describe the covariate-relevant population characteristics of the human research participants (e.g. age, genotypic information, past and current diagnosis and treatment categories). If you filled out the behavioural & social sciences study design questions and have nothing to add here, write "See above."*
Recruitment	*Describe how participants were recruited. Outline any potential self-selection bias or other biases that may be present and how these are likely to impact results.*
Ethics oversight	*Identify the organization(s) that approved the study protocol.*

Note that full information on the approval of the study protocol must also be provided in the manuscript.

Field-specific reporting

Please select the one below that is the best fit for your research. If you are not sure, read the appropriate sections before making your selection.

☒ Life sciences ☐ Behavioural & social sciences ☐ Ecological, evolutionary & environmental sciences

For a reference copy of the document with all sections, see nature.com/documents/nr-reporting-summary-flat.pdf

Life sciences study design

All studies must disclose on these points even when the disclosure is negative.

Sample size	A minimum of 3 samples per condition were used for in vitro quantification analysis and a minimum 8 rats per condition were used for the in vivo studies. No statistical methods were used to predetermine sample size.
Data exclusions	No sample was excluded for this study. Single cells were filtered using the criteria detailed in the manuscript.
Replication	In vitro experimental findings were reproducible in at least three biological or independent experiments. All n values are stated for all experiments in the figure legends.
Randomization	In vitro samples were allocated based on genotype.
Blinding	All the animal studies were performed by blinded investigators and investigators who performed HPLC were blinded to the methodologies used for midbrain dopaminergic differentiation. Investigators who performed in vitro quantification analysis were not blinded, however they followed a semi-automatic object-based quantification analysis, where all the images were analyzed with conserving binary object segmentation settings.

Reporting for specific materials, systems and methods

We require information from authors about some types of materials, experimental systems and methods used in many studies. Here, indicate whether each material, system or method listed is relevant to your study. If you are not sure if a list item applies to your research, read the appropriate section before selecting a response.

Materials & experimental systems

n/a	Involved in the study
☐	☒ Antibodies
☐	☒ Eukaryotic cell lines
☒	☐ Palaeontology and archaeology
☐	☒ Animals and other organisms
☒	☐ Clinical data
☒	☐ Dual use research of concern
☒	☐ Plants

Methods

n/a	Involved in the study
☒	☐ ChIP-seq
☐	☒ Flow cytometry
☒	☐ MRI-based neuroimaging

Antibodies

Antibodies used

goat anti-OTX2 (1:500, R&D Systems, cat# AF1979), mouse anti-CDX2 (1:200, BioGenex, cat# MU392-UC), mouse anti-Engrailed1 (EN1, 1:40, DSHB, cat# 4G11-s), rabbit anti-EN1 (1:50, Merck, cat# HPA073141), rabbit anti-FOXA2 (1:500, Cell Signaling, cat# 8186), goat anti-FOXA2 (1:200, R&D Systems, cat# AF2400), rabbit anti-LMX1A (1:5000, Millipore, cat# AB10533), mouse anti-TH (1:2000, Millipore, cat# MAB318), rabbit anti-TH (1:1000, Pel Freez, cat# P40101-150), chicken anti-MAP2 (1:2500, Abcam, ab92434), rabbit anti-GIRK2 (1:500, Alomone, cat# APC-006), mouse anti-CALB1 (1:5000, SWANT, cat #300), rabbit anti-Collagen3A1 (1:1000, NovusBio, cat# NB120-6580), sheep anti-hCOL1A1 (1:200, R&D Systems, cat# AF6220), mouse anti-HNA (1:200, Abcam, cat# ab191181), rabbit anti-GABA (1:1000, Sigma-Aldrich, cat#A2052) and mouse anti-hSYP (1:750, Enzo LifeSciences, cat#ADI-905-782-100). 5-HT (1:15,000, Sigma-Aldrich, S5545).
For FACS, FOXA2 (1:300, PE-conjugated, Miltenyi Biotec), OTX2 (1:300, VioB515-conjugated, Miltenyi Biotec), EN1 (1:50, Atlas Antibodies), and LMX1A (1:2500, Novo Nordisk).

Validation

The antibodies used in this study have been used in previously published reports and/or validated by manufacturer.

Eukaryotic cell lines

Policy information about cell lines and Sex and Gender in Research

Cell line source(s)

H9 (WA09) and H1 (WA01) cell lines, obtained from WiCell. DANi002C (https://hpscreg.eu/cell-line/DANi002-C)

Authentication

Fingerprinting analysis, immunostaining for pluripotent markers.

Mycoplasma contamination

All cell lines tested negative for mycoplasma contamination.

Commonly misidentified lines
(See ICLAC register)

N/A

Animals and other research organisms

Policy information about studies involving animals; ARRIVE guidelines recommended for reporting animal research, and Sex and Gender in Research

Laboratory animals

9 week old NIH (NTac:NIH-Foxn1rnu) nude male rats purchased from Taconic Biosciences A/S. For the second in vivo experiment 6-8 week old male rats were purchased from Charles River (Crl:NIH-Foxn1rnu).

Wild animals

N/A

Reporting on sex

Adult male rats were used in this study. To avoid variation in animal size we chose only male rats.

Field-collected samples

N/A

Ethics oversight

All animal experiments were conducted in accordance with the guidelines of the European Union Directive (2010/63/EU) and approved by the Danish Animal Inspectorate.

Note that full information on the approval of the study protocol must also be provided in the manuscript.

Flow Cytometry

Plots

Confirm that:

☒ The axis labels state the marker and fluorochrome used (e.g. CD4-FITC).

☒ The axis scales are clearly visible. Include numbers along axes only for bottom left plot of group (a 'group' is an analysis of identical markers).

☒ All plots are contour plots with outliers or pseudocolor plots.

☒ A numerical value for number of cells or percentage (with statistics) is provided.

Methodology

Sample preparation	For sorting of cells to generate clonal cell line, the cells were dissociated with Accutase to obtain single cells and resuspended in cell culture media and sorted on a FACSAriaIII.<br>For flow cytometry analysis, cells cryopreserved at 16 DIV were thawed, and dead cells were labeled with a fixable near-infrared viability dye (1:1000, Invitrogen) in neurobasal medium (Gibco) supplemented with 1% N2 supplement (Gibco) for 15 minutes at room temperature protected from light. The cells were then washed once with FACS buffer (1% bovine serum albumin in PBS-/-), pelleted by centrifugation at 400g for 10 minutes, and fixed and permeabilized using the Transcription Factor Buffer Set (BD Biosciences) according to the manufacturer's instructions. For each sample, 0.5 x 106 fixed cells were incubated with a cocktail of primary antibodies in Perm/Wash buffer (BD Biosciences) for 30 minutes at 4°C protected from light. The cells were then washed three times with Perm/Wash buffer, pelleted by centrifugation at 800g for 3 minutes, and incubated with a cocktail of secondary antibodies for 30 minutes at 4°C protected from light. Finally, the cells were washed twice with Perm/Wash buffer, pelleted by centrifugation at 800g for 3 minutes, resuspended in 200 µL of FACS buffer, and passed through a 40 µm cell strainer (Merck Millipore). Compensation was performed using compensation beads (anti-REA Compensation Beads, Miltenyi Biotec; UltraComp eBeads™ Compensation Beads, Invitrogen; ArC Amine Reactive Compensation Kit, Invitrogen). 20,000 live cell events were recorded per sample.
Instrument	NovoCyte Quanteon analyzer (Acea Biosciences Inc., Santa Clara, CA), FACSAriaIII (BD Biosciences, San Jose, CA), and CytoFLEX S flow cytometer (Beckman Coulter, Indianapolis, IN, USA)
Software	NovoCyte Quanteon analyzer (Acea Biosciences Inc., Santa Clara, CA), CytExpert software, FlowJo software (v10.7.2, Ashland, OR, USA)
Cell population abundance	For the generation of GBX2 knockout line and 4X cell line produced from single-cell sorting, the purify of clones were analysed by whole exome sequencing. For flow cytometry analysis at least 5,000 counts were recorded.
Gating strategy	Gating was based on FSC/SSC, debris were removed by gating on the main cell population. Singlets were gated by FSC-A/FSC-H. Positive population were gated compared to a negative control.

☒ Tick this box to confirm that a figure exemplifying the gating strategy is provided in the Supplementary Information.
